# Creation and Annihilation of Skyrmions in the Frustrated Magnets with Competing Exchange Interactions

**DOI:** 10.1038/s41598-017-16348-8

**Published:** 2017-11-22

**Authors:** Yong Hu, Xiaodan Chi, Xuesi Li, Yan Liu, An Du

**Affiliations:** 1 College of Sciences, Northeastern University, Shenyang, 110819 China; 2 Physics Department, University of California, Davis, California, 95616 USA

## Abstract

In triangular-lattice magnets, the coexistence of third-neighbor antiferromagnetic and nearest-neighbor ferromagnetic exchange interactions can induce rich magnetic phases including noncoplanar skyrmion crystals. Based on Monte Carlo simulation, we studied the dependence of magnetic phase transition on exchange interaction strength. Under the consideration of uniaxial anisotropy and magnetic field both perpendicular to the film plane, a large antiferromagnetic exchange interaction induces a high frustration. When the value of antiferromagnetic exchange interaction is one and a half times larger than the ferromagnetic one, a magnetic phase composed of canting spin stripes, never observed in the chiral magnets, forms. Interestingly, different canting spin stripes along three 120 degree propagation directions may coexist randomly in a magnetic phase, attesting that the canting spin stripes are three-fold degenerate states akin to helices and the multiple state of canting spin stripes is a circular configuration with zero skyrmion charge number. Moreover, skyrmions and antiskyrmions can be observed simultaneously in the configuration at the low temperature nearly close to 0 K, and their configuration and diameter properties are discussed. Finally, the mechanisms of skyrmion creation and annihilation are properly interpreted by comparing exchange and Zeeman energy terms.

## Introduction

Magnetic frustration, namely the energies of all pairs of magnetic interactions in a system that cannot be minimized simultaneously, may arise from the competition of several magnetic interactions, the coupling of several degrees of freedom (spin and orbit), the coupling of several order parameters (elastic, magnetic and electric) in ordered systems or come from the geometrical or topological constraints^1–5^. Although the spin orientations in frustrated systems are disordered at the temperatures well below the Curie-Weiss point, there has been growing interest in investigating mechanisms to generate order in frustrated systems^6^. It has been reported that the frustrated magnets can form a new class of materials that can host skyrmions^7–10^. Magnetic skyrmions as localized noncollinear states tend to crystallize predominantly in a hexagonal lattice form (or sometimes in a tetragonal or cubic lattice form). Experimentally, the skyrmions can be observed either in the reciprocal space using small-angle neutron scattering (SANS)^11^ or in the real space by virtue of Lorentz transmission electron microscopy (LTEM)^12–16^.

Skyrmions are promising candidates as information carriers for future information-processing devices due to their small sizes^11–13,17^, the motion with ultralow current density (~100 A·cm^−2^)^11,18^ and the emergent electromagnetic properties induced by their topological nature^19,20^. Moreover, Sampaio *et al*.^21^ demonstrated that an individual skyrmion can be a stable configuration, locally nucleated by injection of spin-polarized current, and displaced by current-induced spin torques even in the presence of large defects. Subsequently, Tamasello *et al*.^22^ compared the technological advantages and limitations of using Bloch- and Néel-type skyrmions, and presented that the Néel skyrmion moved by the spin Hall effect is a very promising strategy for technological implementation of the next generation of skyrmion racetrack memories. The skyrmions with high densities or the skyrmion crystals with short periods are desirable for the emerging spintronic applications^13,16,20^. In spite of the skyrmions appearing as ubiquitous magnetic objects or order in all the non-centrosymmetric B20 transition metal compounds with Dzyaloshinskii-Moriya (DM) interactions, there have been many unsolved issues on the skyrmions created in the centrosymmetric compounds which have the helimagnetic structures arising from other interactions, such as magnetic frustration. Chakraverty *et al*.^7^ presented an experimental report on the three-dimensional skyrmion crystals in SrFeO_3_ and SrFe_0.99_Co_0.01_O_3_ thin films induced by exchange interactions. Theoretically, Hayami *et al*.^8^ studied the easy-axis anisotropy dependence of magnetic structures in frustrated magnets and suggested that a strong anisotropy and competing exchange interactions can together evolve skyrmion crystals into bubble crystals. In the framework of Landau theory, skyrmions and bubbles are both triple-**Q** orderings stabilized by anisotropies, where **Q** is the ordering wave vector to maximize the electronic magnetic susceptibility, and thus the helices propagation direction. Different from skyrmions, the spins in a bubble are parallel and the bubble is not chiral, i.e. with zero skyrmion charge number (*τ* = 0).

In addition, Okubo *et al*.^9^ and Leonov and Mostovoy^10^ in their literatures both predicted the coexistence of skyrmions and antiskyrmions in frustrated magnets due to centrosymmetric nature. Skyrmions and antiskyrmions are distinguished conventionally based on the sign of skyrmion charge number (*τ*). If the magnetic field is positive (i.e. along the + *z*-axis direction), the spin texture with the skyrmion charge number of *τ* = −1 is called skyrmion, while that with the *τ* = +1 skyrmion charge number is called antiskyrmion. The skyrmion charge number of *τ* = ±1 can be used to store information. The sign reversal of skyrmion charge number leads to the opposite direction of the topological Magnus force, and hence the accumulation of skyrmions and antiskyrmions at the opposite edge due to the skyrmion Hall effect, which may be very useful for implementation of logical operations^10,23^. Recently, Nayak *et al*. using LTEM directly observed the room-temperature magnetic configurations in tetragonal Heusler Mn_1.4_PtSn and Mn_1.4_Pt_0.9_Pd_0.1_Sn materials experimentally^24^. Based on the magnetization distributions (configurations) and the LTEM patterns of the deflected electrons, they redefined the Bloch- and Néel-type skyrmions and antiskyrmions. For the Bloch-type skyrmions, a transverse helix with a counterclockwise type spin rotation is evident and a ring-like pattern should appear when imaged them using LTEM. Whereas the magnetization is counterclockwise-rotated in a spin cycloid for the Néel-type skyrmions, and no intensity modulation is expected in the case of the Néel-type skyrmions viewed by LTEM due to a closed loop made by the deflected electrons. In the antiskyrmions, the magnetization rotates both as a transverse helix and as a cycloid, leading to form two bright and two dark lobes in the LTEM image^24^. Because the antiskyrmions break the cylindrical symmetry and have a quadrupolar moment of magnetostatic charges, their properties may differ from those of the Bloch- and Néel-type skyrmions.

In contrast to the DM interactions, the exchange interactions are insensitive to the direction of spin rotation in the noncollinear magnetic states, which gives skyrmions two additional degrees of freedom—vorticity and helicity. By the way, the skyrmion helicity can interact with an applied electric field and the coupling between the helicity and the center-of-mass dynamics of skyrmion leads to a characteristic magnetoelectric effect^25^. Therefore, the skyrmion crystals driven by exchange interactions are much denser than the DM counterpart, and the purely exchange-interaction-driven skyrmion crystals with spontaneously opposite vorticity and helicity may exhibit much more functionalities. However, the exchange interaction constant dependence of magnetic phase transitions of skyrmion crystals has remained elusive. To our knowledge, the skyrmion creation and annihilation in such frustrated magnets have not been predicted yet because the proper experimental samples are scarce and the micromagnetism approximations are difficult to be used on the triangular-lattice magnets. In this paper, an unbiased Monte Carlo simulation is performed to demonstrate the effect of exchange interactions on the magnetic skyrmion phase transitions, the skyrmion configurations, and the skyrmion creation and annihilation in the triangular-lattice magnetically frustrated films. To unravel the mechanism of skyrmion formation in the simplest frustrated magnets will help us to understand and design more skyrmionic devices based on diverse types of competing interactions.

## Results

### Magnetically frustrated films with competing exchange interactions

When a film thickness is reduced to several tens of nanometers, the sample can be safely regarded as a magnetically two-dimensional (2D) system. Therefore, our calculations are mostly carried out on a 2D triangular lattice with *L* ^2^ = 64^2^ spins and a lattice constant of *a*. We have checked the *L* size dependence up to *L* = 200, and no additional effect from the size variation on the magnetic phases and their transitions exists (partial results shown in the Supplementary Information). To induce a twisted magnetic structure, the third-neighbor antiferromagnetic (*j*′) and nearest-neighbor ferromagnetic (*j*) exchange interactions are considered simultaneously. Using the experimental data and mean-field results, Nakatsuji *et al*.^2^ suggested that *j*′ is nearly five times larger than *j* in magnetically frustrated NiGa_2_S_4_. However, in NiBr_2_ *j*′ may be only a quarter of magnitude of *j* approximately^26^. Apparently, the ratio of *j*′ to *j* depends on material species and experimental treatments, and the *j*′/*j* general dependence of magnetic structure has been unclear yet. However, in previous literatures^8,9,11,25,27^, it has been found that the values of *j*′/*j* determine the module of **Q**, i.e.,

1 $$|{\bf{Q}}|=\frac{2}{a}\,{\cos }^{-1}[\frac{1}{4}(1+\sqrt{1+\frac{2j}{j^{\prime} }})].$$

The value of |**Q**| characterizes the degree of helices. Using our model, the skyrmions can be crystallized in a hexagonal lattice form only when *j*′/*j* is larger than 0.6. From Eq. (1), the critical value of *j*′/*j* where the spirals form is calculated 0.25, corresponding to |**Q**| = 0. However, our simulation results suggest that the stripes or labyrinth domains at small magnetic fields can be observed when *j*′ ≥ 0.4*j*. With the increase of *j*′, the skyrmions appear and increase at some fields. They cannot be spontaneously arranged densely into a triangular lattice until *j*′ ≥ 0.6*j* (seen in the Supplementary Information). These discrepancies for small *j*′ between simulation results and Eq. (1) solutions probably arise from the finite temperature and non-zero anisotropy. We regard the skyrmions in dense skyrmion crystals as skyrmion stable state to study their phase transitions and sizes. Therefore, the results of magnetic phases in the film with varied *j*′ from 0.6*j* to 2*j* are displayed hereinafter, where *j* serves as an energy unit. In addition, it has proven that the uniaxial anisotropy (*k*) with an easy axis perpendicular to the film plane can stabilize the skyrmions and *k* = 0.5*j* is considered throughout the paper without loss of generality^28,29^. Lastly, the magnetic field (*h*) is applied along the easy-axis direction. The effect of dipole-dipole interactions on the magnetization behaviors and magnetic phases is discussed in the Supplementary Information, and it is found that the dipole-dipole interactions in this model cannot add or remove any magnetic phases, just make the magnetic phase transitions occur under stronger magnetic fields^30^. Furthermore, in contrast to the results obtained by Ezawa^31^, the dipole-dipole interactions weakly affect the skyrmion sizes in this paper, probably due to the skyrmion crystals induced by strong competing exchange interactions instead of DM interactions. Therefore, the dipole-dipole interactions are neglected in order to save the computational time. Hence the total Hamiltonian of the system can be expressed as

2 $$H=-j\sum _{ < i,m > }{{\bf{S}}}_{i}\cdot {{\bf{S}}}_{m}+j^{\prime} \sum _{ <  < i,n >  > }{{\bf{S}}}_{i}\cdot {{\bf{S}}}_{n}-k\sum _{i}{({S}_{i}^{z})}^{2}-g{\mu }_{{\rm{B}}}h\sum _{i}{S}_{i}^{z},$$

where **S** denotes the unit vector of classical Heisenberg spin, *g* the Landé *g*-factor, *μ*_B_ the Bohr magneton, <*i*, *m*> and <<*i*, *n*>> the summations over the nearest-neighbor and third-neighbor pairs only.

### The *j*′ dependence of *h*-induced magnetic phase transition and the *j*′-*h* magnetic phase diagram

**Figure 1:**
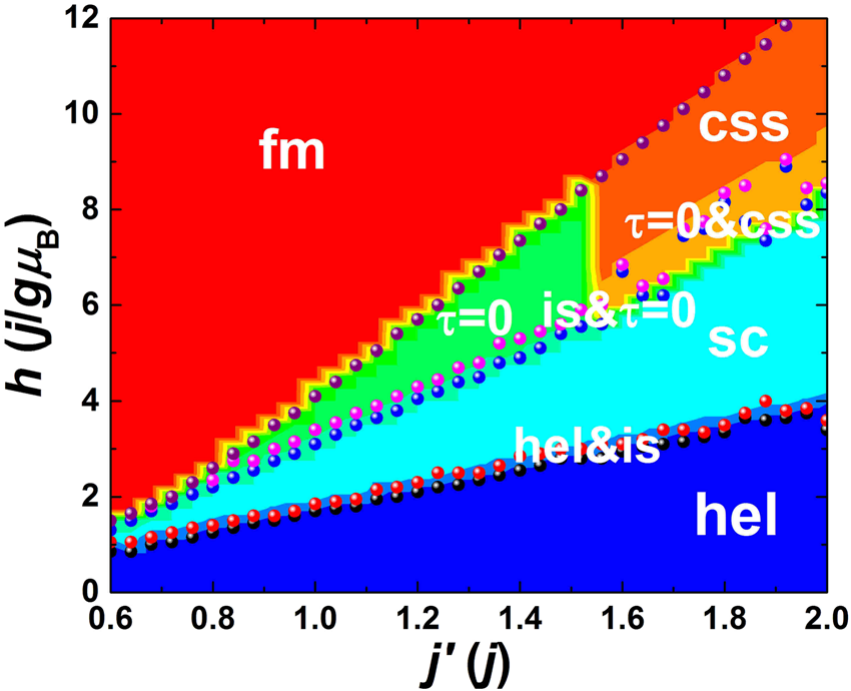
Magnetic phase diagram with the increase of magnetic field and antiferromagnetic exchange interaction. Different magnetic phases are identified including helices (hel), skyrmion crystals (sc), individual skyrmions (is), *τ* = 0-state configurations (*τ* = 0), canting spin stripes (css), ferromagnetic state (fm), and their mixed phases. The phases are distinguished using different colors. The boundaries between neighbor phases shown by spheres are determined by analyzing the results of spin configuration, magnetic susceptibility and specific heat.

In many previous literatures^8,9,11–13^, the magnetic phases at different temperatures and magnetic fields or at different anisotropies and magnetic fields are characterized and classified into a figure called the magnetic phase diagram. The magnetic phase diagram provides a complete evolution picture of the magnetic structures with adjustable parameters, advantageous for understanding of the dependence of specified magnetic phases on adjustable parameters. In this paper, we aim to demonstrate the *j*′ dependence of magnetic phases, especially the skyrmion phases, and thus the *j*′-*h* magnetic phase diagram is delineated in Fig. 1 firstly. This *j*′-*h* magnetic phase diagram is created by classifying the spin configurations at different *j*′ and *h*, and the phase boundaries are doubly checked by magnetic susceptibility and specific heat results.

The configurations in different phases will be described and discussed in the next subsection. In the absence of magnetic field, the magnetic phase of helices (hel) is observed for all studied *j*′, and this phase is kept under small magnetic fields. With the increase of magnetic field, the helices (hel) go way to skyrmion crystals (sc) through a mixed phase composed of helices (hel) and individual skyrmions (is). The magnetic field of phase transition is roughly linearly proportional to *j*′. Moreover, as shown in Fig. 1, the magnetic field range of skyrmion crystals (sc) is wider for larger *j*′, and the width increases also roughly linearly with *j*′. It is implied that both the magnetic phases of helices (hel) and skyrmion crystals (sc) are stabilized by *j*′, and the stability also depends on the magnetic field. Significantly, with the further increase of magnetic field, the magnetic phase type created after skyrmion crystals (sc) is determined by *j*′. When 0.6*j* ≤ *j*′ < 0.8*j*, the skyrmion crystals (sc) directly go way to ferromagnetic (fm) state. If *j*′ is varied from 0.8*j* to 1.55*j*, the magnetic phase of the so-called *τ* = 0-state configurations (*τ* = 0)^32^ forms from skyrmion crystals (sc) through a mixed phase of individual skyrmions (is) and *τ* = 0-state configurations (*τ* = 0). For 1.55*j* < *j*′ ≤ 2*j*, the magnetic phase transitions under strong magnetic fields become complicated. The skyrmion crystals (sc) firstly go way to the mixed phase of individual skyrmions (is) and *τ* = 0-state configurations (*τ* = 0). Then the magnetic phase may randomly go into the other mixed phase of *τ* = 0-state configurations (*τ* = 0) and different canting spin stripes (css, will be interpreted later), followed by a uniform canting spin stripes (css) phase. In other words, the mixed phase composed of different canting spin stripes (css) may appear only in a narrow range of magnetic fields or even does not appear at some *j*′, resulting in no clear phase boundary between skyrmion crystals (sc) and canting spin stripes (css). Finally, the ferromagnetic (fm) state is observed under strong enough magnetic fields, and the magnetic field at the onset of ferromagnetic (fm) state is also linearly proportional to *j*′ with a higher slope. The appearance and transition of magnetic phases at different *j*′ and magnetic fields are interpreted in the next subsection.

### The spin configurations of magnetic phases and the chirality properties of skyrmion crystals

**Figure 2:**
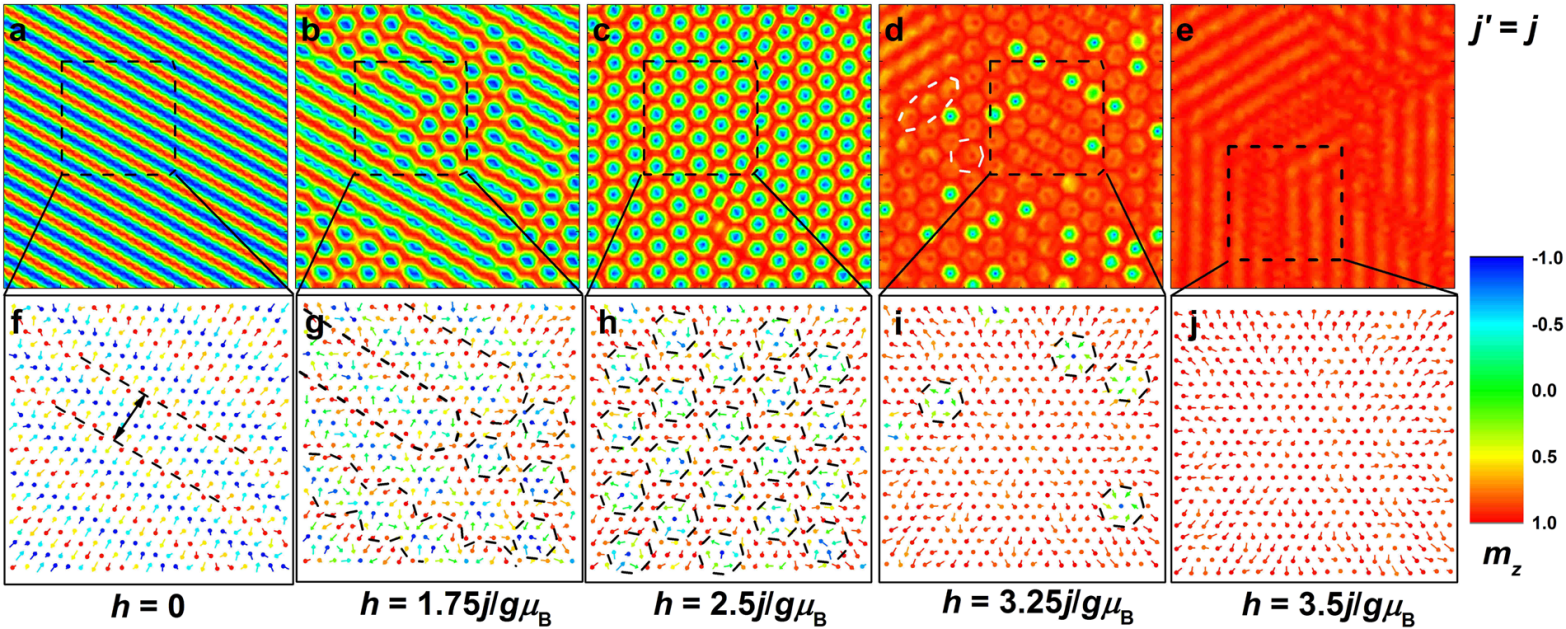
Real-space magnetization distribution and the spin configuration in the selected area in the film with *j*′ = *j* under selected magnetic fields. The color scale is the value of out-of-plane component of magnetization. White dashed rings in d indicate the *τ* = 0-state (*τ* = 0) magnetization distribution. The period of helices (hel) is shown in f. The magnetic phases of merons, skyrmion chains, skyrmion crystals (sc), individual skyrmions (is), and *τ* = 0-state configurations (*τ* = 0) are also indicated in g–i.

**Figure 3:**
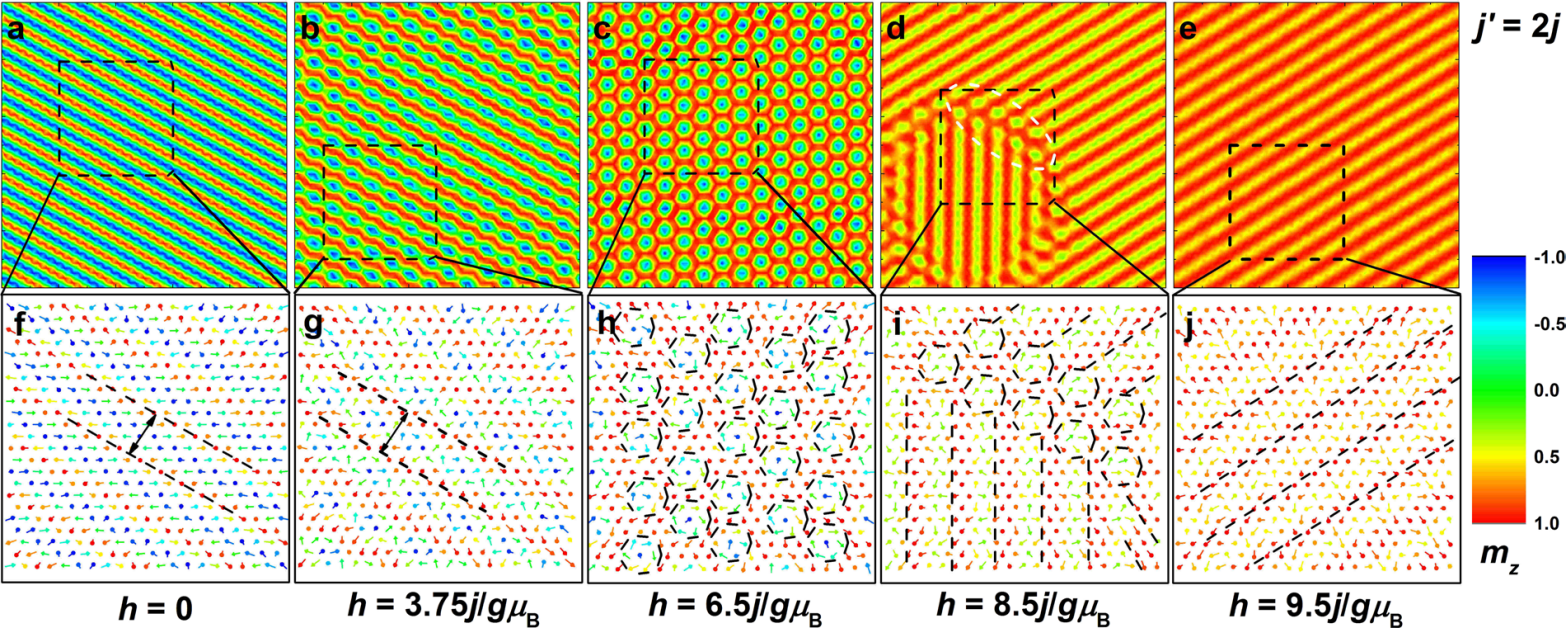
Real-space magnetization distribution and the spin configuration in the selected area in the film with *j*′ = 2*j* under selected magnetic fields. The color scale is the value of out-of-plane component of magnetization. White dashed ring in d indicates the magnetization distribution of the connection between *τ* = 0-state configurations (*τ* = 0) and canting spin stripes (css). The period of helices (hel) that is independent of magnetic field is shown in f,g. The skyrmion crystals (sc) are displayed in h. The mixed magnetic phase of *τ* = 0-state configurations (*τ* = 0) and canting spin stripes (css) along different propagation directions are given in i. The uniform canting spin stripes (css) are shown by parallel dashed lines in j.

In this subsection, the real-space microscopic spin configurations in different magnetic phases with different *j*′ are studied. In particular, the chirality of skyrmion crystals (sc) and thus the skyrmion density with different *j*′ is calculated. The skyrmions and antiskyrmions are found to exist simultaneously in a spin configuration. At first, the real-space spin configurations in the film with *j*′ = *j* and 2*j* under selected magnetic fields are showed in Figs 2 and 3, respectively. When the magnetic field is absent, the spin configuration of helices (hel) is observed. The positive and negative out-of-plane components of magnetization are equidistant, and the helices (hel) period for *j*′ = *j* is wider than that for *j*′ = 2*j*, as indicated in Figs 2a,f and 3a,f. The 2D triangular lattice itself provides a spatial anisotropy to align the helices (hel) along the three equivalent directions, and these directions for each spin just point to its next-neighbor or third-neighbor spins under 120 degrees, depending on which ones couple to it antiferromagnetically^33^. In this paper, the three equivalent directions are superimposed on the three exchange bonds under 120 degrees. The helices (hel) along the three directions are degenerate in energy and only one of the three equivalent directions is chosen randomly. Thus the magnetic phase of helices (hel) is topologically trivial single-**Q** state^33,34^. On the other hand, from Figs. 2f, 3f, the spins rotate from the positive to negative *z*-axis direction in a plane nearly parallel to the helices (hel) propagation direction, i.e. along the **Q** direction. When the exchange and anisotropy energies are minimized, no spin orientation is perpendicular to the helices (hel) propagation direction, also supporting the fact that the helices (hel) propagation direction is along one of the exchange bonds.

The noncollinear effects of isotropic *j*′ and *j* on the spins experiencing anisotropy make the spin wave propagate in the film plane to construct a noncoplanar magnetic structure. We also keep in mind that the noncollinear exchange interactions induce magnetic frustration, and apparently the spin configuration driven by frustration may be long-range ordered. The ferromagnetic (*j*) exchange interactions encourage the parallel (collinear) spin arrangement, so do magnetic field and anisotropy. However, when the antiferromagnetic (*j*′) exchange interactions are added, the collinear spin alignment will bear the highest antiferromagnetic exchange energy. Thus the collinear spin structure is destabilized for *j*′ ≠ 0 and according to Eq. (1) the minimum value of *j*′ at the onset of helices (hel) should be 0.25*j* without any anisotropy, temperature, and magnetic field. On the other side, if *j*′ increases to infinity, the maximum value of |**Q**| in Eq. (1) can be calculated 2 cos^−1^(0.5)/*a*, that is, the maximum twisted angle between the nearest-neighbor spins equals 120°. Therefore, the twisted configuration is intensified monotonically with the increase of *j*′. In the film without in-plane boundaries, the spin wave can propagate infinitely in the film plane, resulting in smaller periods of helices (hel) for larger *j*′. Under the interplay between *j*′ and *j*, the angle between the nearest-neighbor spins along the **Q** direction should be identical, that is, the helices (hel) should be circular, while under the consideration of anisotropy with the easy axis perpendicular to the film plane, the optimal helices (hel) is elliptical instead, in consistence with the discussion by Hayami *et al*.^8^.

With the increase of magnetic field, the equidistant distribution of positive and negative out-of-plane components of magnetization is broken. The positive component is widened while the negative component is squeezed. Thus the energy barriers to stabilize the helices (hel) become low, and if the spins rotate over the energy barriers, the configuration of multiple-**Q** state may form. If the energy of the primary propagation direction keeps the lowest, the metastable configuration of merons or skyrmion chains should form^35^. When the three amplitudes of **Q** are equal, the skyrmion configuration forms. The magnetic field where the magnetic phase transition occurs and the twisted degree in the skyrmion structure are both dependent on *j*′. In a skyrmion, the spins at the periphery align with the magnetic field, on the contrary, the spins at the skyrmion core are pointing opposite to the magnetic field. From the skyrmion periphery to the skyrmion core, the spins rotate in a plane perpendicular or parallel to the **Q** direction. If the spin spiral propagates by the helical (or cycloidal) way in a skyrmion, the skyrmion is Bloch- (or Néel-) type^22,36^. Furthermore, the range of the magnetic field under which the skyrmion crystals (sc) are sustained depends on *j*′ as well, and the range is wider for larger *j*′. When *j*′ is large, the antiferromagnetic exchange energy in the skyrmion is low (dominant), and the skyrmion spins are trapped by large skyrmion energy barriers as a result of the large *j*′. In order to overcome the high skyrmion energy barriers and finally melt the skyrmions, a much stronger magnetic field is needed for larger *j*′. Therefore, for a large value of *j*′ the values of the magnetic field to stabilize the skyrmion crystals (sc) are not only large but also their range is wide.

The skyrmion can induce one flux quantum of an effective magnetic field acting on spin-polarized electrons and magnons, which gives rise to topological Hall effect in charge and heat transport, and at the same time sets the skyrmion into motion^11,18,21,22^. For these features, the skyrmion chirality properties and the skyrmion sizes with *j*′ are studied. Using the spin configuration of skyrmion crystals (sc), the local chirality (*τ*_*i*_) at site *i* is calculated by

3 $${\tau }_{i}={{\bf{S}}}_{i}\cdot ({{\bf{S}}}_{i+x}\times {{\bf{S}}}_{i+x/2,i+y/2})+{{\bf{S}}}_{i}\cdot ({{\bf{S}}}_{i-x/2,i+y/2}\times {{\bf{S}}}_{i-x})+{{\bf{S}}}_{i}\cdot ({{\bf{S}}}_{i-x/2,i-y/2}\times {{\bf{S}}}_{i+x/2,i-y/2}).$$

**Figure 4:**
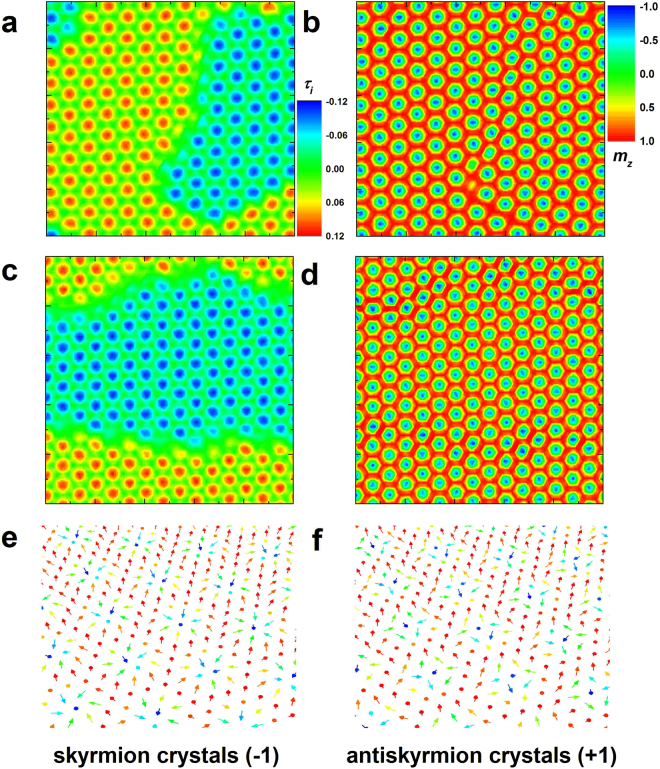
The *in-situ* simulation measurement of real-space chirality and magnetization distributions. The color scale in (**a**,**c**) is the value of local chirality and the color scale in (**b**,**d**–**f**) is the value of out-of-plane component of magnetization. (**a**,**b**) The results obtained in the film with *j*′ = *j* and (**c**,**d**) *j*′ = 2*j* are given. (**e**,**f**) The configurations of skyrmion and antiskyrmion crystals defined by the skyrmion charge number of −1 and +1 are presented, respectively.

**Figure 5:**
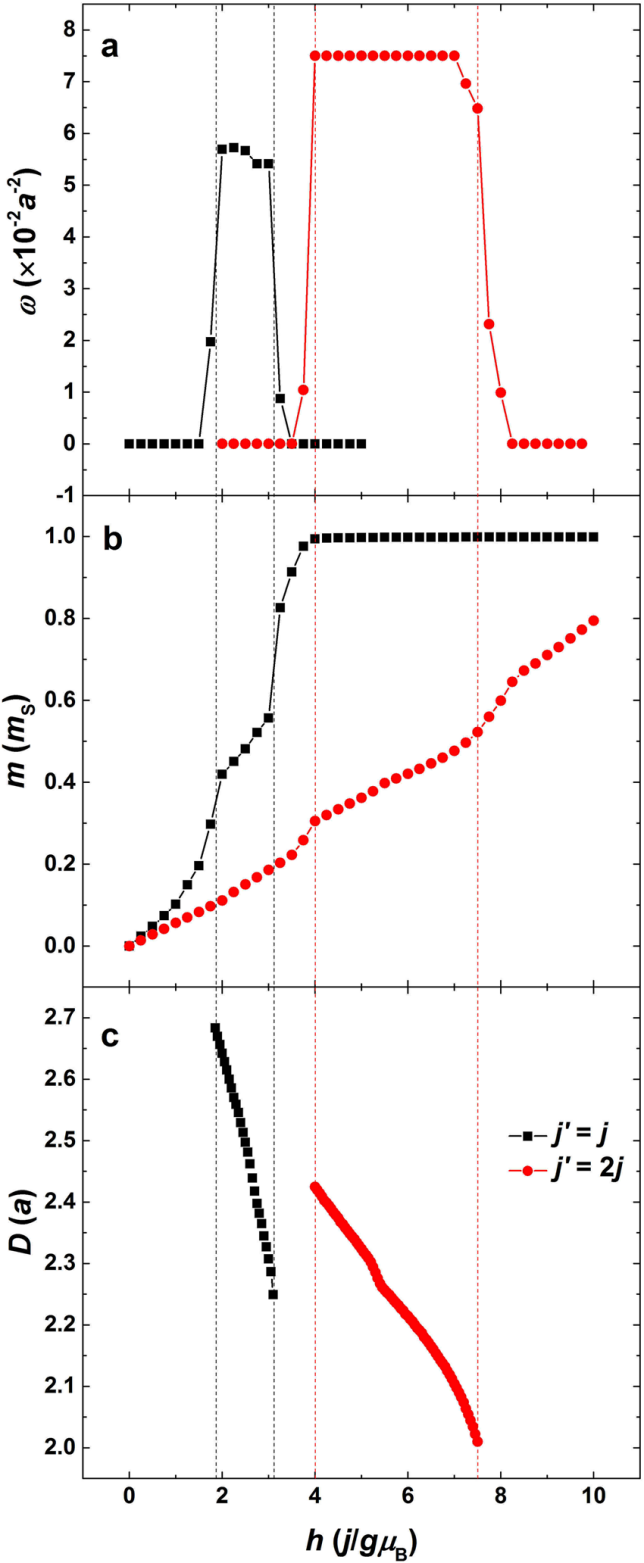
Skyrmion density, out-of-plane component of magnetization, and skyrmion diameter as functions of magnetic field in the film with *j*′ = *j* and 2*j*. The vertical dot lines are the magnetic phase boundaries of skyrmion crystals (sc).

Therefore, the total chirality of a skyrmion (i.e. skyrmion charge number) $\tau =c{\sum }_{i}{\tau }_{i}$ is equal to unity in the continuum limit, where *c* is a constant to grant the unit topological charge of one skyrmion^28,30^. Moreover, the skyrmion density of skyrmion crystals (sc) is calculated by $\omega =\sum |\tau |/A$ where *A* is the lattice area. The spin configuration and the local chirality of skyrmion crystals (sc) in the same area for *j*′ = *j* and 2*j* are both given in Fig. 4a–d and the skyrmion density result is shown in Fig. 5a. Both the negative and positive values of local chirality are obtained, attesting that skyrmions and antiskyrmions coexist^9^. However, the sum over skyrmions and antiskyrmions is less than the skyrmion number obtained from the configuration due to the cancellation between skyrmions and antiskyrmions with opposite equal chirality when they contact. Based on the conservation law of topological nature, they also should be added up to calculate the skyrmion density. In ref.^9^, Okubo *et al*. reported a *Z* configuration composed of skyrmions and antiskyrmions. However, they cannot present the skyrmion configuration feature due to high thermal fluctuations. In this paper, we adjust the magnetic parameters and observe the similar configuration at low or even zero temperature (as discussed in the Supplementary Information). The configuration of skyrmion shown in Fig. 4e indicates that the spins rotate in the radial planes from the periphery to the core, i.e. the skyrmion is Néel-type, similar to the skyrmion type stabilized by interfacial DM interactions^22^. On the other hand, the configuration of antiskyrmion is also well displayed in Fig. 4f and the cross-sections along four different directions reveal both helicoid and cycloid spin propagations in an antiskyrmion, with the similar structure found in chiral magnets^24,37^.

Taking advantage of the results shown in Fig. 4, we can calculate the skyrmion density (*ω*) with the increase of magnetic field for different *j*′. In order to obtain the information of skyrmion size, we resort to the analytic result of skyrmion perimeter per unit area based on the **Q**-state theory presented in ref.^8^,

4 $$\frac{{P}_{s}}{A}=\frac{{3}^{1/4}Q\sqrt{1-M}}{2\sqrt{\pi }}.$$

Although Hayami *et al*. in their literature^8^ presented Eq. (4) when they studied the skyrmions in the similar system, they cannot give an exact result of Eq. (4) due to the unknown magnetization analytically. Further, in order to calculate the skyrmion diameter (*D*), Eq. (4) should be dealt with by

5 $$D=\frac{{P}_{s}}{A}\cdot \frac{1}{\varpi \pi }=\frac{{3}^{1/4}Q\sqrt{1-M}}{2{\pi }^{3/2}\varpi },$$

where $\varpi$ is the averaged skyrmion density in the skyrmion crystals (sc). In Eq. (5), the skyrmion diameter apparently depends on *j*′, magnetization, and skyrmion density. The magnetization is a function of magnetic field. Therefore, the analytic **Q**-state theory is incomplete to calculate the skyrmion diameter. In this paper, the magnetization result can be obtained by means of Monte Carlo simulation, with the results presented in Fig. S4 in the Supplementary Information, and thus Eq. (5) can be solved by combining the results of the **Q**-state theory and Monte Carlo simulations. The skyrmion density and diameter as functions of magnetic field for *j*′ = *j* and 2*j* are both delineated in Fig. 5. The skyrmion density in the skyrmion crystals (sc) exhibits a plateau, and the height of plateau only depends on *j*′. The skyrmion density is larger for larger *j*′, which is also the reason why the skyrmion crystals (sc) for large *j*′ are stable over a wide range of magnetic field. On the contrary, the skyrmion diameter is both influenced by *j*′ and magnetic field. The skyrmion diameter decreases monotonically with the increase of magnetic field, and the decrease of diameter is steep for small *j*′. The magnetic field dependence of skyrmion diameter is similar to that found in DM-interaction-driven multilayers experimentally^38^. All in all, the value of *j*′ determines the twisted degree between neighbor spins, and thus controls the skyrmion size. On the other hand, although the skyrmion spin texture in the skyrmion crystals (sc) is stable with the increase of magnetic field and the skyrmion density is also independent of magnetic field, the magnetic field can drive the spins at the skyrmion periphery locally to align with the field direction. For small *j*′, the antiferromagnetic exchange coupling is weak and thus the magnetic response (susceptibility) is large. So with the increase of magnetic field, the skyrmion diameter for small *j*′ decreases significantly.

In the skyrmions under strong magnetic fields, the spins at the skyrmion core bear high Zeeman energies, and these spins may flip abruptly when the magnetic field is strong enough, resulting in the formation of the *τ* = 0-state configurations (*τ* = 0), where the central core polarity is in parallel alignment with the surrounding spins outside the skyrmions^32^. For *j*′ = 2*j* the skyrmion is broken from the core and the skyrmion periphery simultaneously when the magnetic phase transition occurs. Under a strong magnetic field, the large antiferromagnetic exchange interactions associated with small ferromagnetic exchange interactions even can overcome the magnetic field to twist the spin configuration akin to the magnetic phase of helices (hel). Different from the low-magnetic-field helices (hel), only the positive out-of-plane component of magnetization exists due to strong magnetic fields, and the spins rotate in this twisted configuration not continuously, analogous to the stripe domain alternately aligned with saturated and unsaturated states. We call this stripe domain canting spin stripes (css), which have been presented by Hayami *et al*.^8^. They defined this phase multiple-**Q** conical spirals and did not interpret further. Unfortunately, we cannot agree with that due to an interesting finding not presented in their literature. As shown in Fig. 3d,i, the canting spin stripes (css) along different propagation directions may be observed simultaneously in a configuration and the *τ* = 0-state configurations (*τ* = 0) are just the terminations of the canting spin stripes (css). Therefore, the circular *τ* = 0-state configurations (*τ* = 0) are the multiple-**Q** state akin to skyrmions, whereas the canting spin stripes (css) should be single-**Q** states akin to helices (hel). Furthermore, the magnetic phase composed of *τ* = 0-state configurations (*τ* = 0) and canting spin stripes (css) with different propagation directions appear with probabilities, that is, they may be not observed for some *j*′. As discussed above, the states of spin configurations are degenerate in energy when the spins rotate continuously (helices, hel) or discontinuously (canting spin stripes, css) along the three propagation directions (exchange bonds) under 120 degrees. If they coexist in a spin configuration, their energies are nearly equal. In other words, the spin orientations at the terminations where different canting spin stripes (css) are connected (as indicated by white dashed ring in Fig. 3d) should satisfy the requirements of energy minimization of different propagation directions at the same time. That is to say, the spins at these terminations are hardly trapped by the energy barriers. The complicated magnetic phase is metastable and sensitive to *j*′ and magnetic field, and consequently may appear randomly, which may be also the reason why they are not observed in ref.^8^. On the contrary, the uniform canting spin stripes (css) appear more easily (as seen in Fig. 3e,j).

### The mechanism of skyrmion creation and annihilation

**Figure 6:**
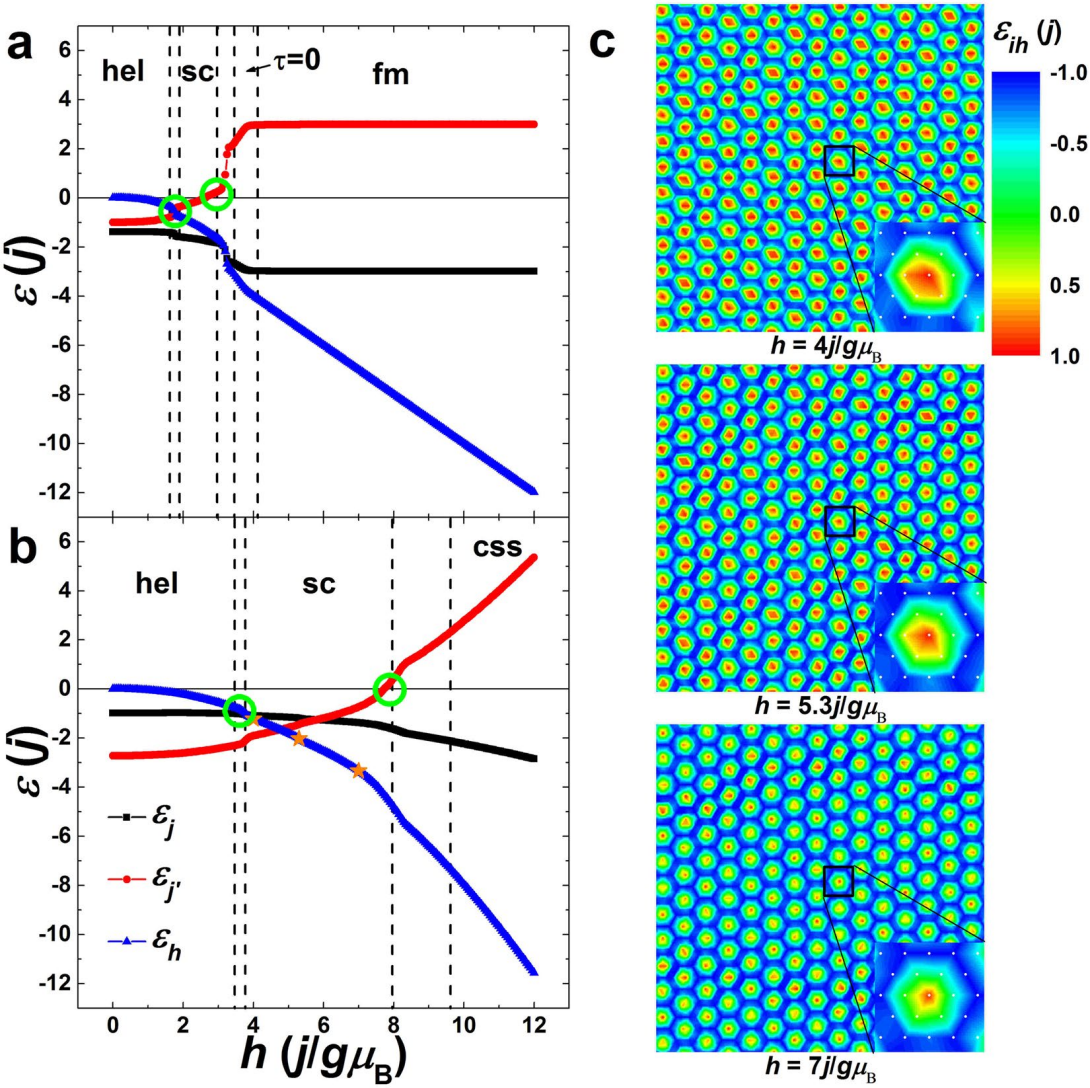
Exchange and Zeeman energy densities as functions of magnetic field. (**a**,**b**) The results obtained in the film with *j*′ = *j* and 2*j* are given, respectively. The intersections of exchange and Zeeman energies and the zero antiferromagnetic exchange energy corresponding to the magnetic phase transitions of skyrmion crystals (sc) are indicated by green rings. The vertical dashed lines separate different magnetic phases. (**c**) The real-space distributions of spin Zeeman energy under the selected magnetic fields indicated by orange stars in (**b**) are presented. Zoomed-in views of the spin Zeeman energy in a skyrmion under different magnetic fields are also shown in (**c**). The color scale is the normalized value of spin Zeeman energy.

In chiral magnets, one has suggested that skyrmion creation and annihilation should occur when the DM-interaction/magnetic-field energy gain is exceeded^12,36,39,40^ and the easy-axis anisotropy is also important in stabilizing the skyrmions^29^. Similarly, the competing exchange and Zeeman interactions should together determine the magnetic phase transitions in the present paper, while their individual roles have not been elucidated yet. In this subsection, we focus on the mechanism to create and annihilate the skyrmions in exchange-interaction-driven frustrated magnets, and the exchange and Zeeman energy densities for *j*′ = *j* and 2*j* as functions of magnetic field are shown in Fig. 6a,b, respectively. When *j*′ = *j* (small *j*′), the ferromagnetic exchange interactions are dominant and the ferromagnetic exchange energy is lower than the antiferromagnetic one. When the magnetic field is applied, the magnetic polarization further stabilizes the ferromagnetic order, while overcomes the antiferromagnetic exchange energy. Thus the Zeeman and ferromagnetic exchange energies are reduced while the antiferromagnetic exchange energy increases with the increase of magnetic field. When the Zeeman energy is lower than the antiferromagnetic exchange energy, the magnetic field is strong enough to trigger the first-order magnetic phase transition from helices (hel) to skyrmion crystals (sc). It is indicated that the helices (hel) are stabilized by the competing exchange interactions, and when the antiferromagnetic exchange energy increases to exceed the Zeeman energy, the antiferromagnetically coupled spin structure is no longer stable. A new configuration is needed to reach the balance where the Zeeman and exchange energies are minimized again, and the topologically protected skyrmions begin to appear. With the further increase of magnetic field, the antiferromagnetic exchange energy increases monotonically. When the antiferromagnetic exchange energy changes their sign from negative to positive, the first-order magnetic phase transition from skyrmion crystals (sc) to *τ* = 0-state configurations (*τ* = 0) is observed. We have discussed that the antiferromagnetic exchange interactions are used to induce the twisted configuration in this system. When the antiferromagnetic exchange energy is equal to zero, the antiferromagnetic exchange energy of each spin may be equal to zero or the antiferromagnetic exchange energies of different spins may add up to zero. For small *j*′, the spins at the skyrmion core may rotate from the negative direction to the in-plane direction to make their orientations perpendicular to their third neighbors, which cancels out the antiferromagnetic exchange interactions to deconstruct the skyrmions.

For *j*′ = 2*j* (large *j*′), the helices (hel) under zero or weak magnetic field are still induced by the competing exchange interactions. At this point, the antiferromagnetic exchange interactions play a primary role and the antiferromagnetic exchange energy is lower. Hence the Zeeman energy will first intersect with the ferromagnetic exchange energy to break the balance of helices (hel) and trigger the magnetic phase transition from helices (hel) to skyrmion crystals (sc). Subsequently, the Zeeman and exchange energies intersect with each other with the increase of magnetic field, whereas the magnetic phase of skyrmion crystals (sc) keeps stable. The magnetic phase transition from skyrmion crystals (sc) to canting spin stripes (css) cannot happen until the antiferromagnetic exchange energy equal to zero, the same as the case for *j*′ = *j*. In Fig. 5, we have observed that the skyrmion diameter decreases with the increase of magnetic field in the magnetic phase of skyrmion crystals (sc). In Fig. 6c, we study the spin Zeeman energy variation in a skyrmion with magnetic field. Apparently, the area of high Zeeman energy at the core in a skyrmion shrinks with the increase of magnetic field. In contrast, the area of zero Zeeman energy increases in the skyrmion, implying that more spins rotate to the in-plane direction to cut off the coupling with magnetic field, also supporting the fact that the magnetic phase of skyrmion crystals (sc) is stable in a wide range of magnetic field for large *j*′.

**Figure 7:**
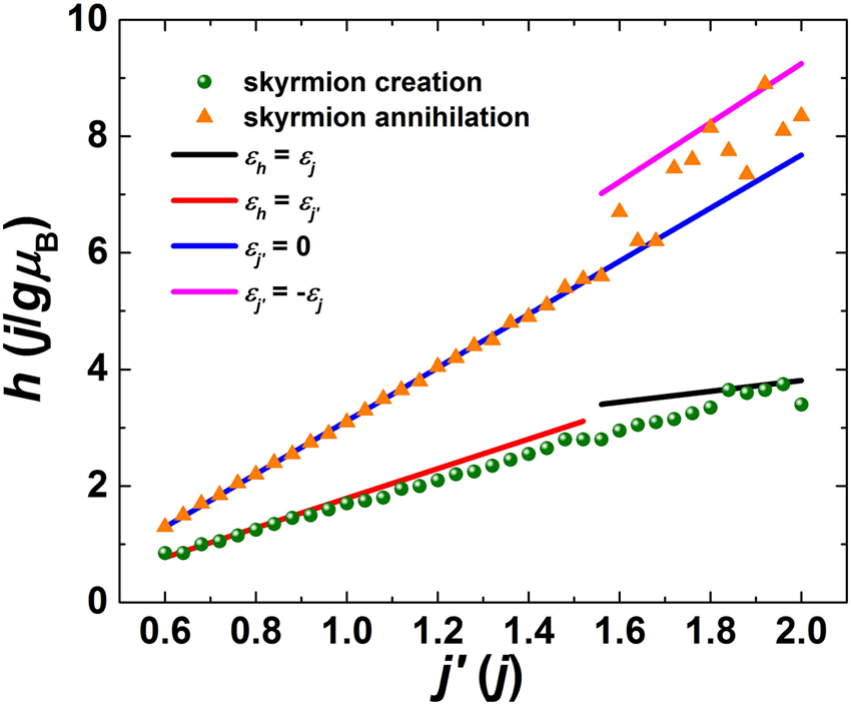
Critical magnetic field as a function of antiferromagnetic exchange interaction. Data points present the magnetic field values where the skyrmion is created (green spheres) or annihilated (orange triangles). Lines present the magnetic field values where the Zeeman and exchange energies intersect or the antiferromagnetic exchange energy is equal to zero or the absolute value of the ferromagnetic exchange energy.

Finally, we summarize the result of magnetic energy variation when the skyrmion is created and annihilated for all studied *j*′, and the critical field result is presented in Fig. 7. Based on the value of *j*′, two correspondences between magnetic energy variation and skyrmion creation/annihilation are obtained. When 0.6*j* ≤ *j*′ < 1.55*j*, the skyrmion is created when the Zeeman energy is equal to the antiferromagnetic exchange energy. The magnetic field of zero antiferromagnetic exchange energy corresponds to the skyrmion annihilation. For the case of 1.55*j* < *j*′ ≤ 2*j*, the skyrmion creation is determined by the Zeeman energy equal to the ferromagnetic exchange energy. On the other hand, for some values of *j*′, the skyrmion annihilation is still observed under the magnetic field where the antiferromagnetic exchange energy is equal to zero. However, for some other values of *j*′, the correspondence between skyrmion annihilation and zero antiferromagnetic exchange energy is no longer valid. The skyrmion may be annihilated corresponding to higher antiferromagnetic exchange energies, which have an upper limit equal to the absolute value of the ferromagnetic exchange energy. From the result shown in Fig. 6c, we have observed that the skyrmion core and periphery may be isolated by in-plane spins. Combined with the skyrmion diameter results shown in Fig. 5, the spins at the skyrmion core are ferromagnetically coupled to each other, while the antiferromagnetic coupling between them and the spins outside of the skyrmion may be cut off. The small ferromagnetic domain forms at the skyrmion core encircled by a circular in-plane magnetic domain^41^. The magnetic field to flip these spins at the skyrmion core needs to overcome the ferromagnetic exchange energy.

## Discussion

In the Monte Carlo simulations, some initial, randomly chosen state of the system is selected and then one proceeds through the lattice determining the change in energy of the system if the spin is rotated; if the energy is lowered the spin is rotated, otherwise it is left unchanged and one proceeds to the next site. The system is swept through repeatedly, and eventually no spin-flips occur; the system is then either in the ground state or in some metastable state^42^. Using the Monte Carlo Metropolis (at finite temperature) or Glauber (at zero temperature) algorithm after simulated annealing, we obtained the magnetic phases, especially the skyrmion crystals (sc), and their transitions as a function of *j*′/*j* and magnetic field. Interestingly, our simulation findings are different from others^8–10,25^, even though we use the same simulation method on the same 2D triangular lattice systems. Leonov and Mostovoy^25^ considered the triangular lattice with *j* = 1 and the antiferromagnetic next-nearest-neighbor exchange interaction with a strength of 0.5*j* under the magnetic field from 0 to roughly 0.6*j*/*gμ*_B_. As a result, they predicted eight configurations to appear in the zero-temperature phase diagram of *k* and magnetic field, and the boundaries are smooth and no mixed phases are observed. However, the most difference from us is, in their results no phases of the *τ* = 0-state configurations (*τ* = 0) and canting spin stripes (css) are obtained. We attribute this difference to the large *j*′ and magnetic field values selected in our paper. The fierce competition between *j*′ and magnetic field triggered more hidden metastable magnetic phases. Moreover, some high-energy metastable states such as the coexistence of skyrmions and antiskyrmions are maintained. Remarkably, the mixed skyrmion crystals (sc) have a higher energy than a purely skyrmion (or purely antiskyrmion) crystal, so they cannot be stabilized at the ground state, as discussed in ref.^25^. However, the large *j*′ selected in this paper may induce high energy barriers, and the high energy barriers prolong the magnetic relaxation time according to the Arrhenius formulation^43^. If the magnetic relaxation time is longer than the measurement time, the magnetic domain walls with high energies may remain at low or even zero temperature after simulated annealing. Quantum or thermal fluctuations also support the formation of high-energy domain walls, which has been reported in ref.^9^. These metastable states are captured by us in this paper and it is convincing that they may disappear for small *j*′ and/or during proper magnetic training. It may also be the reason why the mixed magnetic phases are observed, more like some experimental results^24^.

To summarize, we employed a magnetically frustrated *j*′*-j* model and studied the *j*′ dependence of magnetic-field-driven phase transition. The skyrmion crystals (sc) are observed for all studied *j*′. The magnetic phase of canting spin stripes (css) is only obtained for large *j*′, and the novel intermediate magnetic phase composed of the canting spin stripes (css) along different propagation directions may be observed. Moreover, the *j*′ dependence of skyrmion chirality and diameter was studied. A number of skyrmions and antiskyrmions are identified to coexist in the magnetic phase of skyrmion crystals (sc). The configuration of skyrmion is Néel-type, and the antiskyrmion is similar to that observed in chiral magnets. The skyrmion diameter decreases monotonically with the increase of magnetic field, and the decrease is steep for small *j*′. Finally, a correspondence between magnetic energy variation and skyrmion creation/annihilation is demonstrated to unravel the mechanism of skyrmion in magnetically frustrated films.

Although a comparison of this theory with experiment is beyond the scope of the work reported here, the conclusion is valid for any *C*_6_ invariant frustrated magnets with competing interactions. Furthermore, the skyrmion creation and annihilation are dependent on the comparison between different magnetic energies, instead of the magnetic energies themselves. In other words, the skyrmion creation and annihilation may be adjustable through applying proper magnetic fields or altering exchange interactions by experimental treatments such as alloying or doping. This work not only provides a glimpse of the large variety of magnetic states that may be expected from the particle-like magnetic objects, but further opens a new vista for the interpretation on the creation and annihilation of topologically protected spin textures, which have so far been mostly explored in chiral materials or interfaces. We also leave it as an interesting future exercise to explore the full three-dimensional magnetic phases and their skyrmion phase transitions, as well as the creation and annihilation of three-dimensional skyrmions in such frustrated magnets.

## Methods

Classical Monte Carlo simulations, which incorporate thermal fluctuations in a nonperturbative manner, are used based on the simulated annealing algorithm^44^. There are two stages involved in the simulation. At the first stage, the system is zero-field-cooled from an initial temperature *T*_0_ = 10*j*/*k*_B_ to a target temperature (=0.95^136^ *T*_0_) where *k*_B_ is the Boltzmann constant. That is to say, in every cooling step the new temperature is reduced by the 5% of the previous one. At the second stage, at the target temperature the magnetic field increases between 0 and 12*j*/*gμ*_B_. At each cooling and magnetizing step, 10^5^ times repeated calculations are performed for thermodynamic quasiequalibrium, and succeeding 10^5^ Monte Carlo steps for averaging the quantities such as magnetization and magnetic energies^45,46^. Finally, the spin configuration is averaged over 20 independent runs, and thus the statistic errors are minimized and do not appear in the figures.

## Electronic supplementary material

Supplementary Information
