# Supplementary material for: Creation and Annihilation of Skyrmions in the Frustrated Magnets with Competing Exchange Interactions
Source: Sci Rep. 2017 Nov 22;7:16079. doi: 10.1038/s41598-017-16348-8 (PMC5700090; doi:10.1038/s41598-017-16348-8)
Supplement: Supplementary file 1 — Supplementary Information [file 41598_2017_16348_MOESM1_ESM.pdf]

Supplementary Information in

Creation and Annihilation of Skyrmions in the Frustrated Magnets with Competing Exchange Interactions

Yong Hu[1,2,*], Xiaodan Chi[1], Xuesi Li[1], Yan Liu[1] & An Du[1]

[1]*College of Sciences, Northeastern University, Shenyang 110819, China.*

[2]*Physics Department, University of California, Davis, California 95616, USA.*

1. **Dependence of magnetization and magnetic phase on dipole-dipole interactions**
2. **Magnetization behaviors at zero temperature**
3. **Magnetization behaviors under different magnetic fields for various *j'***
4. **Field-induced magnetic phase transitions in the films with different sizes and small *j'***

Figure S1-S5

* Correspondence and requests for materials should be addressed to Y.H. (email: huyong@mail.neu.edu.cn)

1. Dependence of magnetization and magnetic phase on dipole-dipole interactions

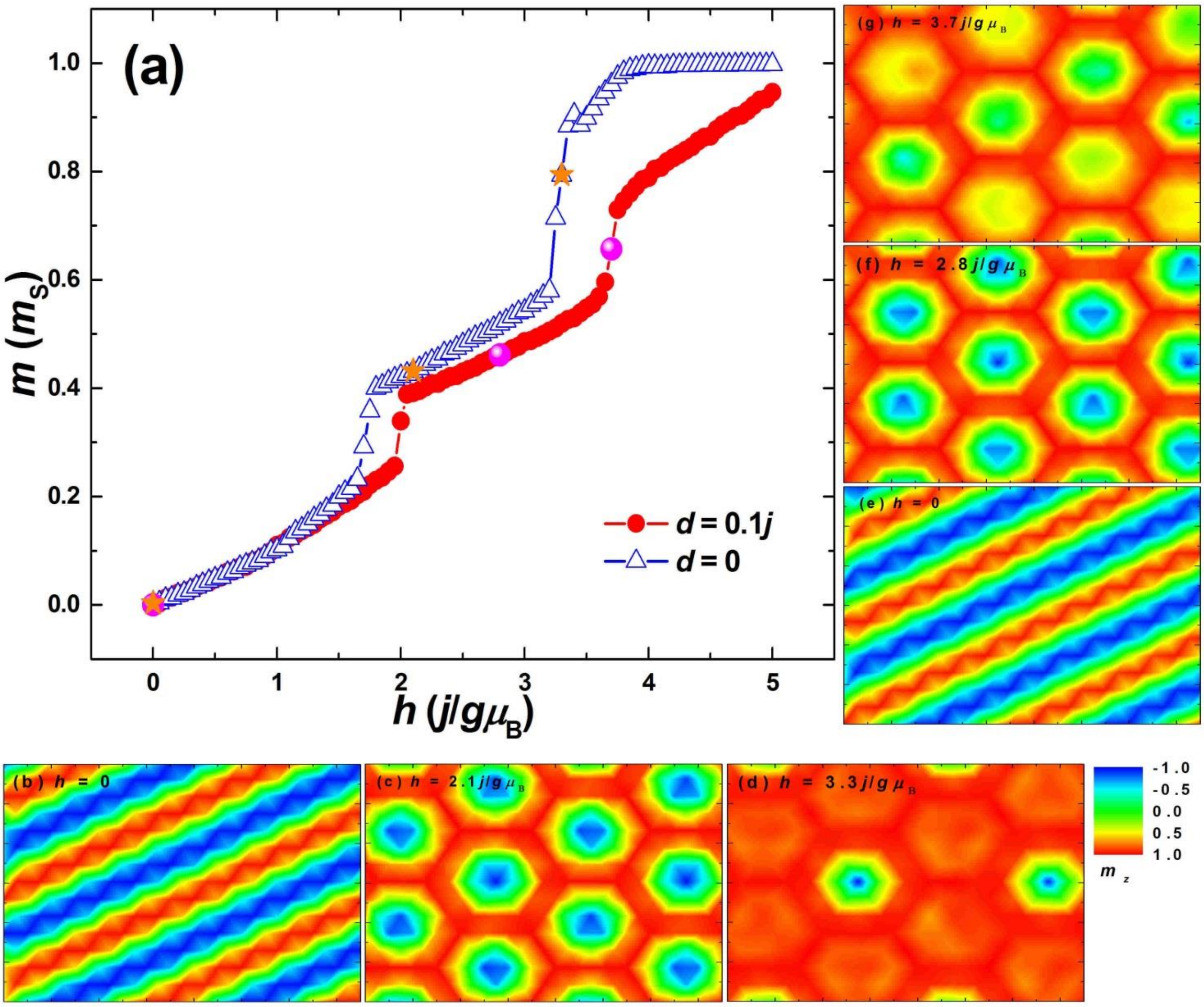

Figure S1. The out-of-plane component of magnetization as a function of magnetic field in the film with and without dipole-dipole interactions and the real-space magnetization distributions under selected magnetic fields for *j'* = *j*. **b-d** are the real-space magnetization results under the selected magnetic fields indicated by orange stars in **a** for the dipole-dipole interactions not taken into account, while **e-g** for the dipole-dipole interactions taken into account indicated by pink spheres in **a**. The color scale is the value of out-of-plane component of magnetization.

In this section, the effect of dipole-dipole interactions on magnetization and magnetic phase transition is discussed. At first, we add the dipole-dipole interactions term,

$$\mathcal{H}_d = d\sum_{i,q}[\frac{\mathbf{S}_i \cdot \mathbf{S}_q}{R_{iq}^3} - \frac{3(\mathbf{S}_i \cdot \mathbf{R}_{iq}) \cdot (\mathbf{S}_q \cdot \mathbf{R}_{iq})}{R_{iq}^5}], \tag{S1}$$

in the Hamiltonian [Eq. (2)] of the text, where the summation is conducted over all the spins in the model, *d* is the dipolar strength constant, $\mathbf{R}_{iq} = \mathbf{r}_i - \mathbf{r}_q$ is the position vector oriented from spin *i* to *q*, $R_{iq} = |\mathbf{R}_{iq}|$ is the distance between spin *i* and *q*. The magnetization behavior and the real-space magnetization distribution in the model with and without dipole-dipole interactions are compared and the results for *j'* = *j* and 2*j* are presented in Fig. S1, S2, respectively. Remarkably, when the dipole-dipole interactions are considered, the magnetization curve under zero and weak magnetic fields is nearly superimposed with that of the dipole-dipole interactions not taken into account. With the increase of magnetic field, the magnetic phase transition occurs from helices (hel) to skyrmion crystals (sc). At the same time, the magnetization curves with and without dipole-dipole interactions are split. With the further increase of magnetic field, the magnetization value under the consideration of dipole-dipole interactions is smaller than that without dipole-dipole interactions. However, from the results of magnetic phase, the magnetic phase transition in the model with dipole-dipole interactions also occurs in sequence from helices (hel), to skyrmion crystals (sc), and then to τ = 0-state configurations (τ = 0) for *j'* = *j*, or to canting spin stripes (css) for *j'* = 2*j*. In other words, the dipole-dipole interactions make the magnetic phases of skyrmion crystals (sc), τ = 0-state configurations (τ = 0) or canting spin stripes (css), and ferromagnetic (fm) states appear under stronger magnetic fields only. The results are in agreement with the simulation results obtained by Du *et al.* using the same method on the Dzyaloshinskii-Moriya-interaction-based model[1].

Magnetic dipole-dipole interactions exist in all magnetic materials, and they are long-range and crystal-shape dependent[2]. They themselves can introduce frustration into a ferromagnetic state to induce a complicated magnetic domain structure in the ground state such as stripes, bubbles, or

labyrinths, which may be the reason why the dipole-dipole interactions can reduce the magnetization under strong magnetic fields, as shown in Fig. S1, S2. Under a proper magnetic field, the dipole-dipole interactions even turn a ferromagnetic state into a giant skyrmion with controllable radius[3]. However, the values of dipole-dipole interactions are generally 100 to 1000 times smaller than the exchange interactions, and the giant (~1-μm-scale) skyrmion driven by the competition between dipole-dipole interactions and exchange interactions also indicates that the magnetic dipole-dipole interactions exhibit a weaker effect on establishing the skyrmions than Dzyaloshinskii-Moriya interactions and exchange interactions. Therefore, we believe that it is still informative when the dipole-dipole interactions are not taken into account in the magnetically frustrated model with fiercely competing exchange interactions, although the dipole-dipole interactions may play a role in the magnetization behavior[1, 3].

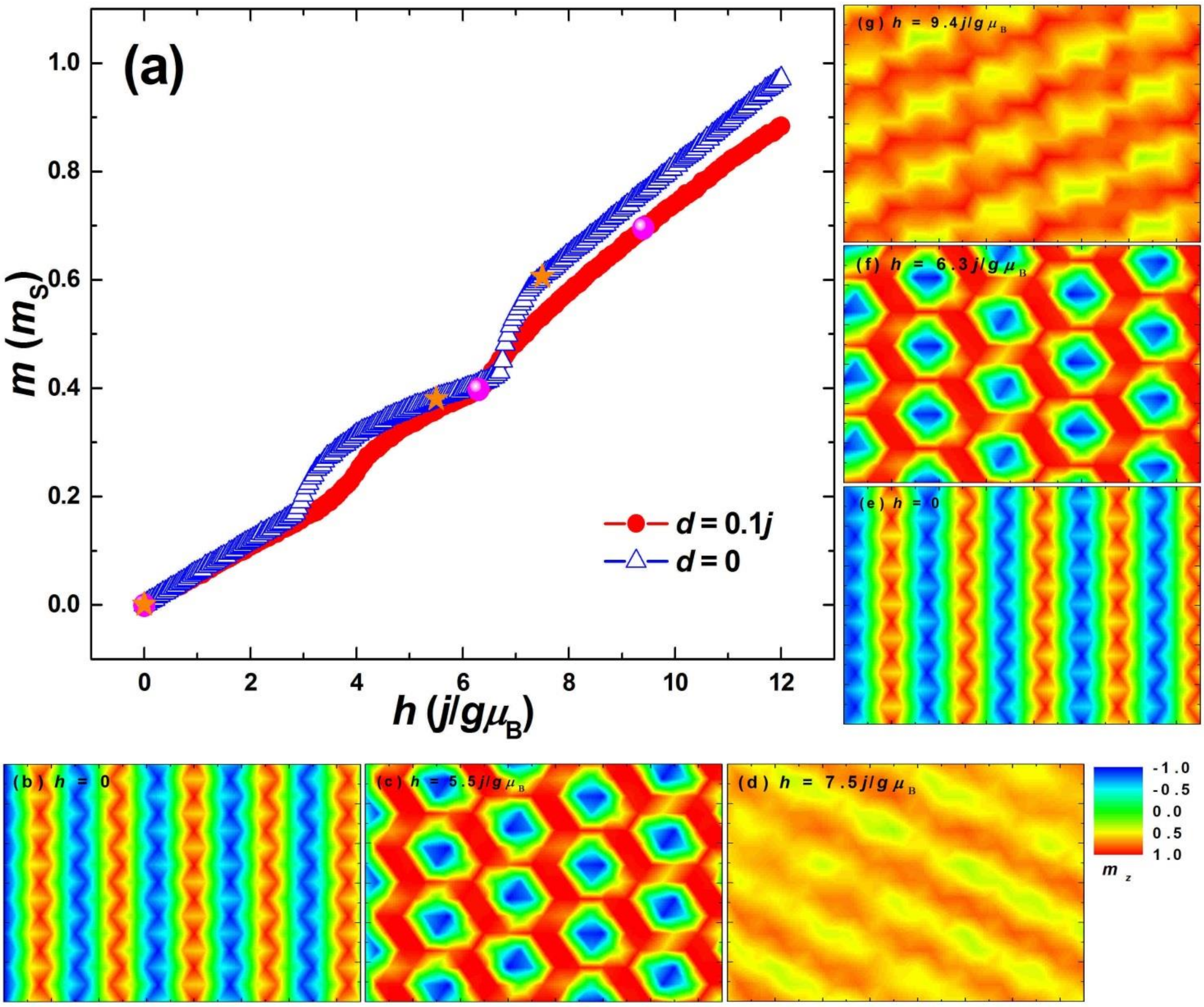

Figure S2. The out-of-plane component of magnetization as a function of magnetic field in the film with and without dipole-dipole interactions and the real-space magnetization distributions under selected magnetic fields for *j'* = 2*j*. b-d are the real-space magnetization results under the selected magnetic fields indicated by orange stars in **a** for the dipole-dipole interactions not taken into account, while **e-g** for the dipole-dipole interactions taken into account indicated by pink spheres in **a**. The color scale is the value of out-of-plane component of magnetization.

2. Magnetization behaviors at zero temperature

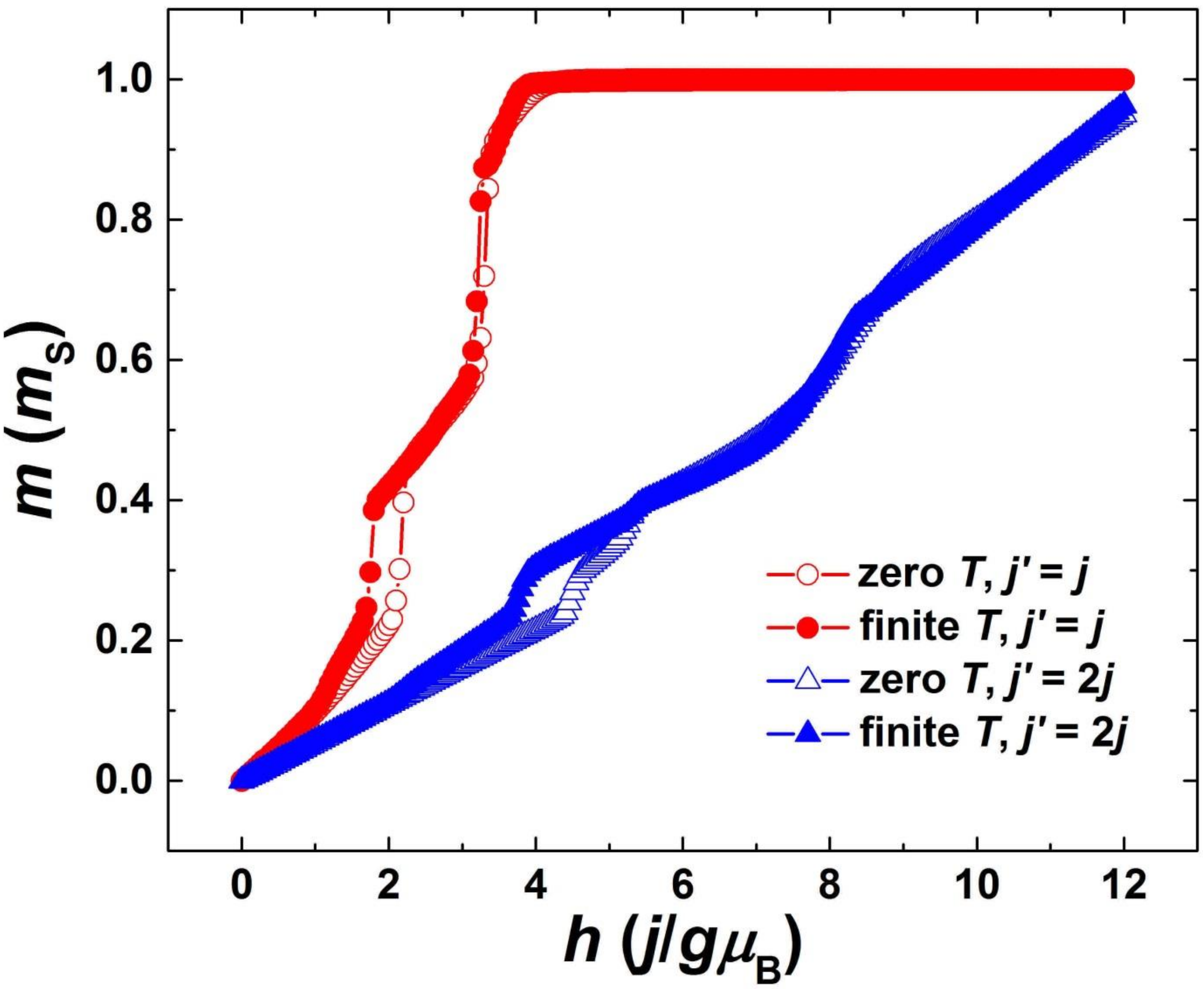

Figure S3. Out-of-plane component of magnetization as a function of magnetic field in the film with *j’* = *j* and 2*j* at zero or finite temperature. The finite temperature value is equal to the temperature value used in the text.

In this section, the magnetization and the spin configuration with the increase of magnetic field in the magnetically frustrated film with *j’* = *j* and 2*j* are both recalculated at zero temperature. For calculating the limit case of zero temperature, the Glauber algorithm is employed[4-6]. One should keep in mind that once the system occurs in a metastable state it will dwell in this state for an arbitrarily long time and this is a frozen metastability approximation. This approach excludes the quantum dynamical effect in that the magnetic system loses the coherence during a very long

relaxation time and the quantum correlation is destroyed. In detail, we first calculate the energy change that would result from flipping a spin. Then, we update the lattice according to the following updating rule: the spin always flips if the energy change is negative, never flips if the energy change is positive, and flips at random if the energy change is zero, i.e., the flip probability is 0.5. The simulation time is also measured by the Monte Carlo step, which is equal to that used in the text.

As shown in Fig. S3, the temperature only determines the magnetic field value where the magnetic phase transition occurs. Moreover, this thermal effect on the magnetic phase transition from helices (hel) to skyrmion crystals (sc) is more significant than that on the magnetic phase transition from skyrmion crystals (sc) to τ = 0-state configurations (τ = 0) or canting spin stripes (css). At finite temperature, the magnetic phase transition occurs under weaker magnetic field than the zero temperature. It is easily acceptable that the thermal fluctuations in the energy competition play a positive role in triggering the magnetic-field-driven first-order magnetic phase transition, which agrees with some suggestions that the temperature can reduce the energy barriers to encourage the spin rearrangement into the topologically protected state arising from Dzyaloshinskii-Moriya interactions or other interactions[7, 8]. It is the reason why a skyrmion or skyrmion crystal (sc) enjoys the topological stability, insofar as the temperature is neither too high nor too low in chiral magnets such as MnSi[7].

Finally, we check the magnetic phase, the skyrmion charge number, and the skyrmion chirality under different magnetic fields at zero temperature, and all the features obtained at finite temperature are observed at zero temperature as well (not shown here). It is attested that the diverse magnetic phases such as skyrmion crystals (sc) and canting spin stripes (css) are intrinsic magnetic phenomena. Interestingly, the conclusion of the skyrmions and antiskyrmions observed simultaneously at zero temperature is contradictory to that drawn by Okubo *et al.* [8] in the similar

magnetically frustrated system. In the model of Okubo *et al.*, the magnetic anisotropy was not considered and the value of j'/j was 3 much larger than ours. Thus we conjecture that it may be attributed to different values of magnetic parameter used between Okubo *et al.* and us.

3. Magnetization behaviors under different magnetic fields for various *j'*

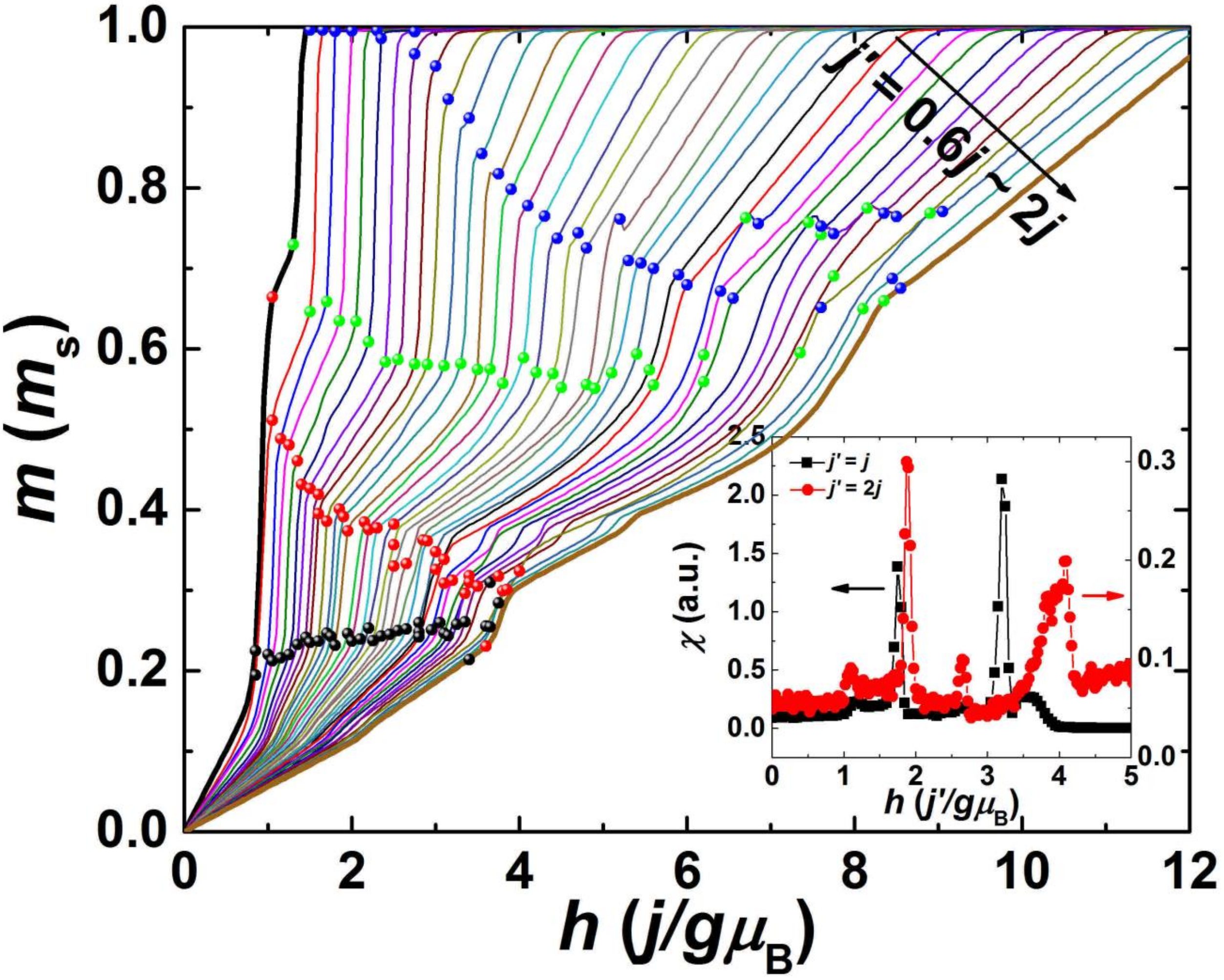

Figure S4. Out-of-plane component of magnetization as a function of magnetic field when *j'* is varied from 0.6*j* to 2*j*. The m_S is the saturated magnetization value. Different phase boundaries are indicated by spheres. Inset gives the selected magnetic susceptibility results.

In Fig. S4, we present the magnetization under different magnetic fields at low temperature for various *j'*. In the absence of magnetic field, there is no net magnetization to be observed. Then the magnetic field is applied and increases towards the +z-axis direction, and as a result, the out-of-plane component of magnetization increases from zero up to saturation. The increase of magnetization with magnetic field also depends on *j'* strongly. The larger the *j'* is, the stronger is the magnetic field needed to saturate the magnetization. Thus, the magnetic field and *j'* are competing

forces.

In addition, the magnetic phases and their phase transitions are identified through analyzing the results of spin configuration, magnetic susceptibility and specific heat as a function of magnetic field (e.g. as shown in the inset of Fig. S4). We study the magnetization behavior and the magnetic phase under different magnetic fields and find that under the magnetic field where the magnetization increases steeply, the magnetic susceptibility should be a delta function approximately. From the spin configuration result, only the magnetic skyrmion phase transition occurs under these magnetic fields. Therefore, the magnetic skyrmion phase transition is first-order.

4. Field-induced magnetic phase transitions in the films with different sizes and small *j'*

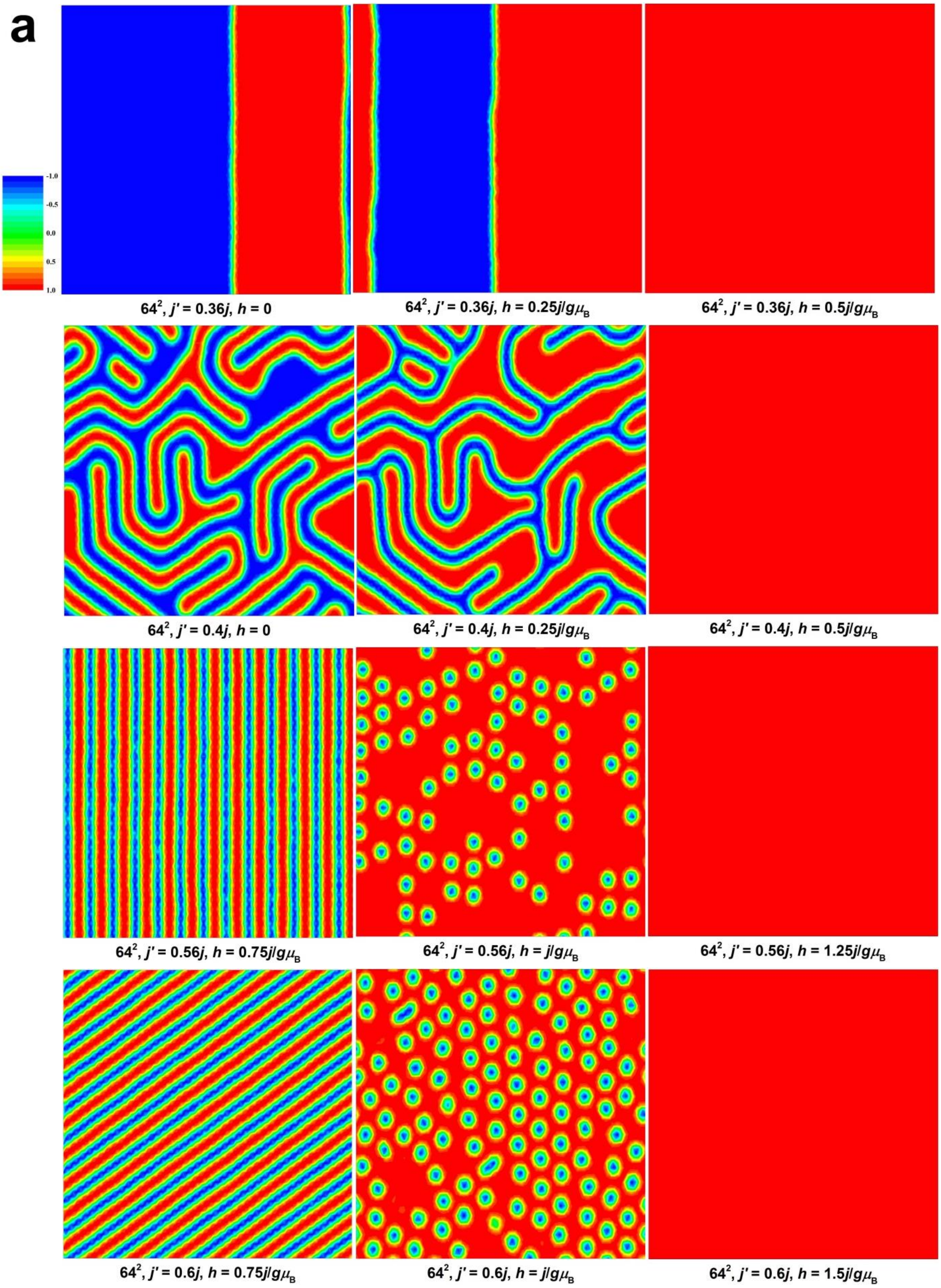

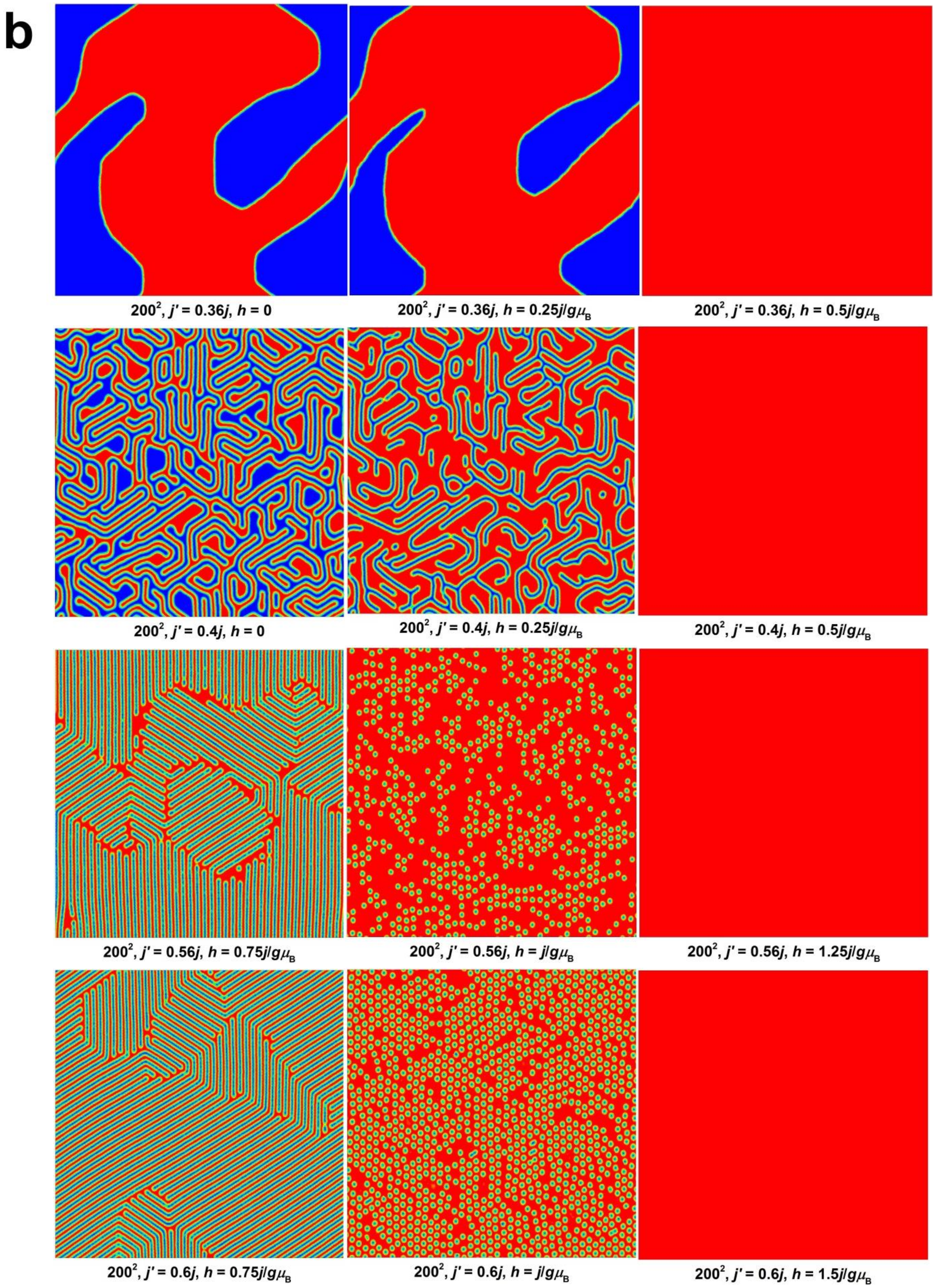

Figure S5. Real-space magnetization distributions in the selected areas in the films with 64^2 and 200^2 spins and different *j'* under selected magnetic fields. The color scale is the value of out-of-plane component of magnetization.

Finally, the magnetic phases and their transitions for small j' are studied in the films with $(64a)^2$ and $(200a)^2$ sizes, and the simulation results are shown in Fig. S5. When $j' < 0.4j$, no spiral configuration is observed, and the large-area 180 degree domains spontaneously form due to anisotropy. This j' value is larger than the calculated value ($0.25j$) obtained from Eq. (1) in the paper. The stripe domains appear as $j' \geq 0.4j$, while the stripes are not straight, i.e., the labyrinth domains form and does not go way to the skyrmion phase with the increase of magnetic field. With the further increase of j', the straight stripe domains increase and the skyrmions may be created by increasing the magnetic field. However, the dense skyrmions, i.e., the skyrmion crystals (sc) are stabilized only when $j' > 0.6j$. The dependence of magnetic phases and their transitions on j' and magnetic field does not change for two sizes.

1. Du, H., Ning, W., Tian, M. & Zhang Y. Field-driven evolution of chiral spin textures in a thin helimagnet nanodisk. *Phys. Rev. B* **87**, 014401 (2013).
2. Chikazumi, S. Physics of ferromagnetism (ed. Chikazumi, S.) 266-273 (Oxford University Press, New York, 2010).
3. Ezawa, M. Giant skyrmions stabilized by dipole-dipole interactions in thin ferromagnetic films. *Phys. Rev. Lett.* **105**, 197202 (2010).
4. Hu, Y., Wu, G., Liu, Y., Yang, X. & Du, A. Defect dependent multiple magnetization plateaus in frustrated spin-chain cobaltate. *J. Magn. Magn. Mater.* **337-338**, 46 (2013).
5. Hu, Y., Shi, F., Jia, N., Liu, Y. & Du, A. Quantitatively microscopic interpretations on magnetization-plateau phenomena in stacked triangular Ising antiferromagnets. *Solid State Commun.* **182**, 5 (2014).
6. Yao, X., Dong, S., Xia, K., Li, P. & Liu, J. M. Spin persistence in an antiferromagnetic triangular Ising lattice under a magnetic field. *Phys. Rev. B* **76**, 024435 (2007).
7. Mühlbauer, S. *et al.* Skyrmion lattice in a chiral magnet. *Science* **323**, 915-919 (2009).
8. Okubo, T., Chung, S. & Kawamura, H. Multiple-q states and the skyrmion lattice of the triangular-lattice Heisenberg antiferromagnet under magnetic fields. *Phys. Rev. Lett.* **108**, 017206 (2012).
